# Maternal and Postnatal High Linoleic Acid Diet Impacts Lipid Metabolism in Adult Rat Offspring in a Sex-Specific Manner

**DOI:** 10.3390/ijms22062946

**Published:** 2021-03-14

**Authors:** Nirajan Shrestha, Josif Vidimce, Olivia J. Holland, James S. M. Cuffe, Belinda R. Beck, Anthony V. Perkins, Andrew J. McAinch, Deanne H. Hryciw

**Affiliations:** 1 School of Medical Science, Griffith University, Gold Coast, QLD 4222, Australia; nirajan.shrestha@griffithuni.edu.au (N.S.); josif.vidimce@griffithuni.edu.au (J.V.); o.holland@griffith.edu.au (O.J.H.); a.perkins@griffith.edu.au (A.V.P.); 2 Institute of Health and Biomedical Innovation, Queensland University of Technology, South Brisbane, QLD 4001, Australia; 3 School of Biomedical Sciences, The University of Queensland, Brisbane, QLD 4072, Australia; j.cuffe1@uq.edu.au; 4 Menzies Health Institute Queensland, Griffith University, Gold Coast, QLD 4222, Australia; b.beck@griffith.edu.au; 5 School of Allied Health Sciences, Griffith University, Gold Coast, QLD 4222, Australia; 6 Institute for Health and Sport, Victoria University, Melbourne, VIC 8001, Australia; andrew.mcainch@vu.edu.au; 7 Australian Institute for Musculoskeletal Science (AIMSS), Victoria University, St. Albans, VIC 3021, Australia; 8 School of Environment and Science, Griffith University, Nathan, QLD 4111, Australia; 9 Environmental Futures Research Institute, Griffith University, Nathan, QLD 4111, Australia

**Keywords:** fetal programming, linoleic acid, lipid metabolism, liver, maternal, postnatal

## Abstract

Linoleic acid (LA), an n-6 polyunsaturated fatty acid (PUFA), is essential for fetal growth and development. We aimed to investigate the effect of maternal and postnatal high LA (HLA) diet on plasma FA composition, plasma and hepatic lipids and genes involved in lipid metabolism in the liver of adult offspring. Female rats were fed with low LA (LLA; 1.44% LA) or HLA (6.21% LA) diets for 10 weeks before pregnancy, and during gestation/lactation. Offspring were weaned at postnatal day 25 (PN25), fed either LLA or HLA diets and sacrificed at PN180. Postnatal HLA diet decreased circulating total n-3 PUFA and alpha-linolenic acid (ALA), while increased total n-6 PUFA, LA and arachidonic acid (AA) in both male and female offspring. Maternal HLA diet increased circulating leptin in female offspring, but not in males. Maternal HLA diet decreased circulating adiponectin in males. Postnatal HLA diet significantly decreased aspartate transaminase (AST) in females and downregulated total cholesterol, HDL-cholesterol and triglycerides in the plasma of males. Maternal HLA diet downregulated the hepatic mRNA expression of *Hmgcr* in both male and female offspring and decreased the hepatic mRNA expression of *Cpt1a* and *Acox1* in females. Both maternal and postnatal HLA diet decreased hepatic mRNA expression of *Cyp27a1* in females. Postnatal diet significantly altered circulating fatty acid concentrations, with sex-specific differences in genes that control lipid metabolism in the adult offspring following exposure to high LA diet in utero.

## 1. Introduction

Lipid accumulation is thought to be due to an imbalance between lipid availability and lipid disposal in the liver as a result of increased intake of certain macronutrients. [1]. Findings from the Dutch Famine study have reported that metabolic diseases may be programmed in utero [2,3]. Maternal obesity and consumption of a high fat diet (HFD) play a significant role in the development of fatty liver and other metabolic disease in offspring [4,5,6]. While increased dietary fat intake by pregnant women is known to be associated with a greater prevalence of the fatty liver disease in offspring, less research has investigated how specific types of fat and fatty acids may be impacting offspring disease. Consumption of the polyunsaturated fatty acid known as linoleic acid (LA, an essential n-6 fatty acid) has increased in recent years and maybe contributing to metabolic disturbances in offspring. Furthermore, once these offspring have started consuming solid food, they are likely to be exposed to the same dietary factors as their mothers and so would be eating large amounts of LA in postnatal life. Given that LA can be metabolised to form inflammatory mediators that are known to be associated with liver injury, [7] it is likely that the combined maternal and offspring exposure to LA may be contributing to the increase in liver disease. Studies have suggested that increased consumption of n-6 PUFA leads to metabolic liver disease, mediated through increased hepatic lipid peroxidation [8,9] however, few studies have explored the role of maternal n-6 PUFA in offspring liver function.

Several lines of evidence reported the developmental origin of lipid dysfunction; however, the potential underlying mechanism is still unknown. The only study to directly investigate the impact of LA on offspring liver function was performed in a rat model which demonstrated that a maternal LA rich diet induces triglyceride accumulation in offspring hepatocytes and alters hepatic lipid metabolism that may predispose the development of liver disease in offspring later in life [10]. While no studies have investigated the mechanism linking high LA to offspring liver disease, we can learn from studies that have investigated how an overabundance of other nutrients might be contributing to offspring metabolic disease. Recently, we have reported an increase in maternal liver weight and changes in inflammatory cytokines in the maternal liver of rats fed with elevated LA [11]. Furthermore, elevated maternal LA results in elevated circulating LA in the fetus [11]. Maternal nutrition and obesity can influence offspring body composition, [12] systolic blood pressure, [13] lipid metabolism [14] and liver disease [4]. However, it is not known if this elevated maternal and postnatal (post-weaning) LA predisposes offspring to alterations in obesity, hypertension, impaired glucose tolerance and dyslipidaemia. 

A number of studies have demonstrated that an adverse postnatal environment can exacerbate disease outcomes and restoration of the postnatal environment may reverse the detrimental effect of maternal diet on offspring health [12,15]. For example, a previous study has shown that a postnatal obesogenic diet exacerbates the adverse effects of maternal obesity [15]. However, consumption of a control diet following maternal HFD consumption during pregnancy did not reverse adverse metabolic disease in her offspring [16]. In addition, postnatal folic acid supplementation has been shown to ameliorate the changes induced by elevated maternal folic acid consumption and led to a reduction in insulin resistance in male offspring but an increase in insulin resistance in female offspring [12]. Given that fetal programming describes how the fetus adapts to the dietary factors it has been exposed, changing the postnatal environment that is different from that experience in utero may contribute to adult-onset of disease. At this time, we do not know if the impact of an elevated maternal LA diet on offspring health could be reversed by a postnatal diet with recommended concentrations of LA or if this may instead exacerbate negative outcomes.

In this study, we aimed to investigate the effects of a maternal high LA (HLA) diet on adiposity, blood pressure, glucose tolerance and parameters associated with lipid metabolism in the liver of adult rat offspring. Furthermore, we investigated whether the postnatal diet could reverse or exacerbate any adverse effects of the maternal diet high in LA. It is well accepted that developmental programming of metabolic diseases occurs in sex-specific manner [17,18], therefore we separately analysed both male and female adult offspring in the present study.

## 2. Results

### 2.1. Effect of Maternal and Postnatal Hla Diet on Body Weight and Organ Weight of Adult Offspring

There was no significant difference in body weight among the groups (Table 1; Supplementary Figure S1). Organ weights were collected and normalised to body weight. The brain weight as a percentage of body weight was significantly increased in male offspring exposed to the postnatal HLA diet (*p* = 0.003). The tibialis anterior (TA; *p* = 0.005) and extensor digitorum longus (EDL; *p* = 0.002) when normalised to body weight were significantly higher in female offspring from dams fed with HLA diet. There was an interaction between the maternal and postnatal diet in the relative left kidney (*p* = 0.03) and soleus (*p* = 0.011) weights compared to total body weight in female offspring. 

### 2.2. Effect of Maternal and Postnatal HLA Diet on Plasma Fatty Acid Composition in Adult Offspring

Maternal and postnatal diet altered FA in male and female offspring (Table 2). Postnatal HLA diet significantly decreased plasma SFA (*p* = 0.003), MUFA (*p* < 0.0001), total n-9 FA (*p* < 0.0001), total n-7 FA (*p* = 0.001), total n-3 PUFA (*p* < 0.0001), ALA (*p* = 0.012), EPA (*p* = 0.04), DPA (*p* = 0.0002) and DHA (*p* < 0.0001) in male offspring (Table 2). The plasma DPA was significantly decreased in male offspring exposed to maternal HLA diet (*p* = 0.019). Postnatal HLA diet significantly increased plasma total n-6 PUFA (*p* < 0.0001), LA (*p* < 0.0001), AA (*p* = 0.0006), LA/ALA (*p* < 0.0001) and AA/DHA (*p* < 0.0001) in male offspring. In female offspring, postnatal HLA diet significantly decreased plasma MUFA (*p* < 0.0001), total n-9 FA (*p* < 0.0001), total n-7 FA (*p* = 0.0005), total n-3 FA (*p* < 0.0001), ALA (*p* = 0.0009), EPA (*p* = 0.014) and DHA (*p* < 0.0001). Maternal HLA diet significantly decreased plasma EPA (*p* = 0.014) and DPA (*p* = 0.04) in female offspring. Postnatal HLA diet significantly increased total n-6 FA (*p* < 0.0001), LA (*p* = 0.006), AA (*p* = 0.002), LA/ALA (*p* < 0.0001) and AA/DHA (*p* < 0.0001) in female offspring. Maternal HLA diet increased LA/ALA (*p* = 0.004) and DHA/DPA (*p* = 0.02) in the plasma of female offspring.

### 2.3. Effect of Maternal and Postnatal Hla Diet on Fat Mass, Lean Mass and Systolic Blood Pressure in Adult Offspring

**Figure 1:**
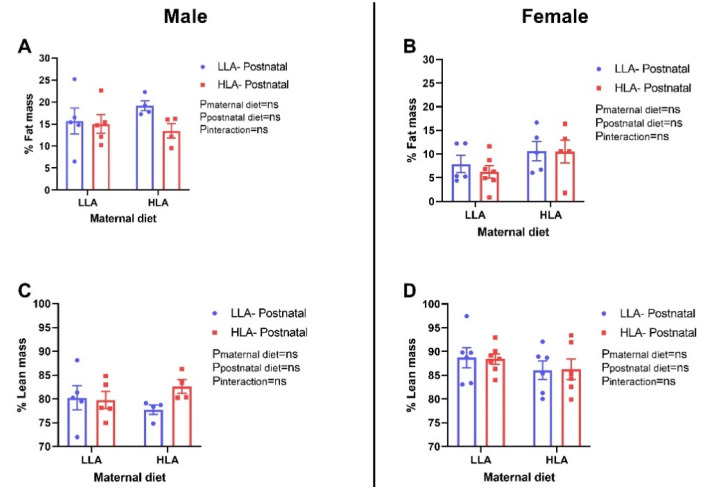
Effect of a maternal and postnatal diet high in linoleic acid on fat mass and lean mass in adult offspring. Data are presented as mean ± standard error of the mean (SEM). Two-way ANOVA was performed for statistical analysis with maternal diet and postnatal diet as two factors. *n* = 4–7. LLA: low linoleic acid; HLA: high linoleic acid; PN: postnatal. **A**: % Fat mass males, **B**: % Fat mass females, **C**: % Lean mass males, **D**: % Lean mass females

**Figure 2:**
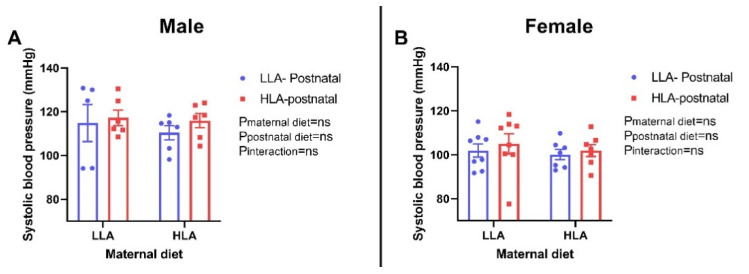
Effect of a maternal and postnatal diet high in linoleic acid on systolic blood pressure of adult offspring. Data are presented as mean ± standard error of the mean (SEM). Two-way ANOVA was performed for statistical analysis with maternal diet and postnatal diet as two factors. *n* = 5–8. LLA: low linoleic acid; HLA: high linoleic acid; PN: postnatal. **A**: Systolic blood pressure males; **B**: Systolic blood pressure females

There were no differences in fat mass and lean mass among the groups in both sexes (Figure 1). Similarly, there were no changes in systolic blood pressure among the groups in both sexes (Figure 2).

### 2.4. Effect of Maternal and Postnatal Hla Diet on Glucose Tolerance and Insulin Tolerance in Adult Offspring

**Figure 3:**
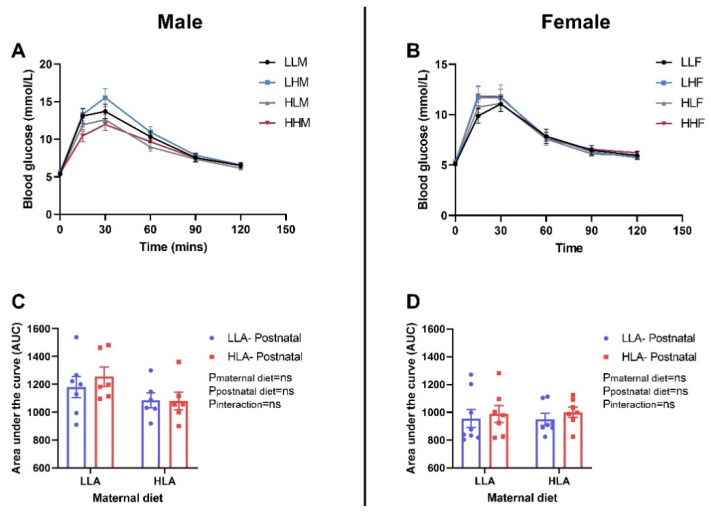
Effect of a maternal and postnatal diet high in linoleic acid on glucose tolerance in adult offspring. Data are presented as mean ± standard error of the mean (SEM). The area under the curve (AUC) was calculated using GraphPad Prism. Two-way ANOVA was performed for statistical analysis with maternal diet and postnatal diet as two factors. *n* = 6–8. LLA: low linoleic acid; HLA: high linoleic acid; PN: postnatal; GTT: glucose tolerance test. **A**: Blood glucose male; **B**: Blood glucose female; **C**: Area under the curve male; **D**: Area under the curve female.

**Figure 4:**
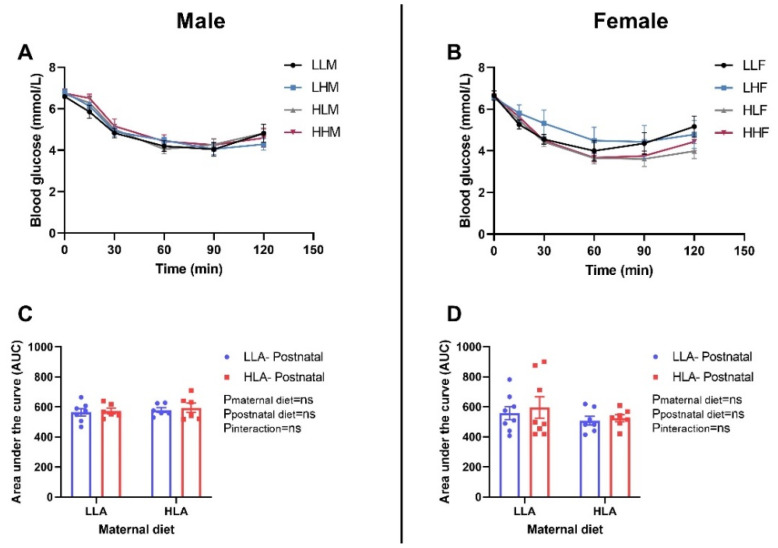
Effect of a maternal and postnatal diet high in linoleic acid on insulin tolerance in adult offspring. Data are presented as mean ± standard error of the mean (SEM). The area under the curve (AUC) was calculated using GraphPad Prism. Two-way ANOVA was performed for statistical analysis with maternal diet and postnatal diet as two factors. *n* = 6–8. LLA: low linoleic acid; HLA: high linoleic acid; PN: postnatal; ITT: insulin tolerance test. **A**: Blood glucose male; **B**: Blood glucose female; **C**: Area under the curve male; **D**: Area under the curve female.

There were no differences in glucose tolerance and insulin tolerance among the groups in both sexes (Figure 3 and Figure 4). Glucose tolerance was estimated by evaluating the blood glucose concentrations in different time points (0, 30, 60 and 120 min) after intraperitoneal injection of glucose. There was no difference in the blood glucose level at each time point among the groups (Figure 3A,B). Insulin tolerance in the adult offspring was evaluated by measuring the blood glucose levels in different time points (0, 30, 60 and 120 min) after intraperitoneal injection of insulin. In both male and female offspring, there was no difference in the blood glucose level at individual time point among the groups (Figure 4A,B).

### 2.5. Effect of Maternal and Postnatal Hla Diet on Circulating Leptin and Adiponectin Concentrations in Adult Offspring

**Figure 5:**
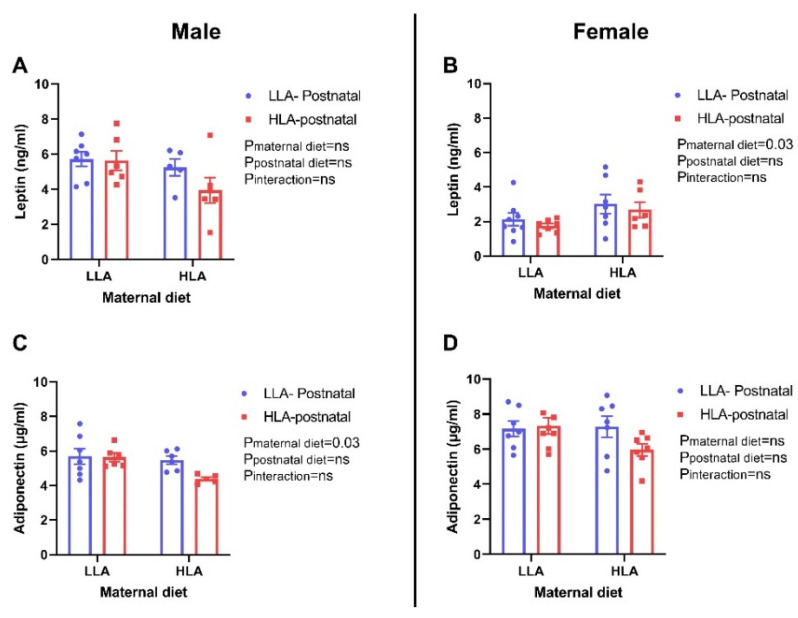
Effect of a maternal and postnatal diet high in linoleic acid on circulating leptin and adiponectin in adult offspring. Data are presented as mean ± standard error of the mean (SEM). Two-way ANOVA was performed for statistical analysis with maternal diet and postnatal diet as two factors. *n* = 5–8. LLA: low linoleic acid; HLA: high linoleic acid; PN: postnatal. **A**: Leptin concentration male; **B**: Leptin concentration female; **C**: Adiponectin concentration male; **D**: Adiponectin concentration female.

Maternal HLA diet significantly increased circulating leptin concentration in female offspring (*p* = 0.03), however, there was no changes in the leptin concentration in male offspring (Figure 5). The circulating concentration of adiponectin was significantly decreased by maternal HLA diet in male offspring (*p* = 0.03), however, it remained unchanged in female offspring (Figure 5).

### 2.6. Effect of Maternal and Postnatal HLA Diet on Circulating Liver Enzymes in Adult Offspring

**Figure 6:**
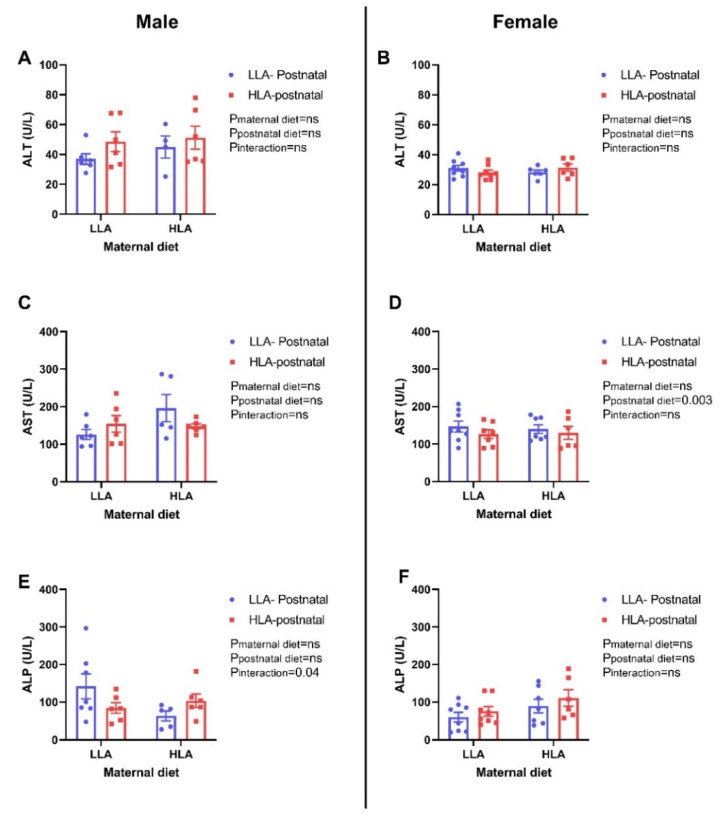
Effect of a maternal and postnatal diet high in linoleic acid on circulating liver enzymes in adult offspring. Data are presented as mean ± standard error of the mean (SEM). Two-way ANOVA was performed for statistical analysis with maternal diet and postnatal diet as two factors. *n* = 5–8. LLA: low linoleic acid; HLA: high linoleic acid; PN: postnatal; ALT: alanine transaminase; AST: aspartate transaminase; ALP: alkaline phosphatase. **A**: ALT concentration male; **B**: ALT concentration female; **C**: AST concentration male; **D**: AST concentration female; **E**: ALP concentration male; **F**: ALP concentration female.

There was no significant difference in circulating alkaline transaminase (ALT) concentration among the groups in both sexes (Figure 6). Postnatal HLA diet significantly decreased aspartate transaminase concentration (AST; *p* = 0.003) in female offspring, however, there was no effect for AST concentrations in male offspring. Maternal and postnatal diet had an interaction effect on circulating alkaline phosphatase (ALP) concentrations with a decrease in male offspring (*p* = 0.04), however, it remained unchanged in female offspring.

### 2.7. Effect of Maternal and Postnatal Hla Diet on Creatinine, Urea and Uric Acid in Plasma of Adult Offspring

**Figure 7:**
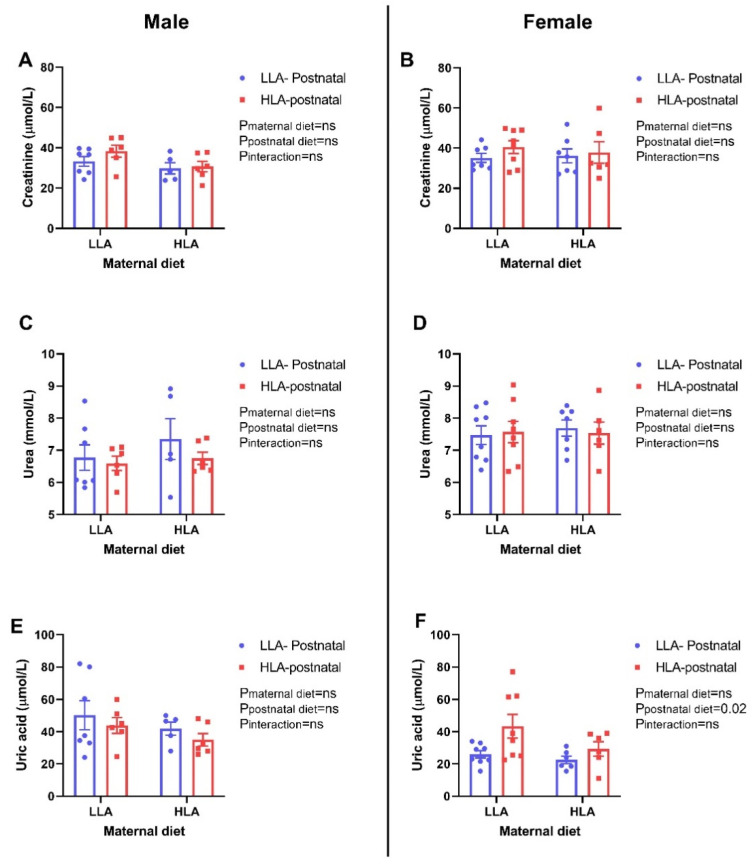
Effect of a maternal and postnatal diet high in linoleic acid on circulating creatinine, urea and uric acid in adult offspring. Data are presented as mean ± standard error of the mean (SEM). Two-way ANOVA was performed for statistical analysis with maternal diet and postnatal diet as two factors. *n* = 5–8. LLA: low linoleic acid; HLA: high linoleic acid; PN: postnatal. **A**: Creatinine concentration male; **B**: Creatinine concentration female; **C**: Urea concentration male; **D**: Urea concentration female; **E**: Uric acid concentration male; **F**: Uric acid concentration female.

There were no changes in circulating creatinine and urea among the groups in both sexes (Figure 7). Postnatal HLA diet significantly elevated the amount of uric acid in the plasma of female offspring (*p* = 0.02), however, there was no difference in the amount of uric acid in male offspring.

### 2.8. Effect of Maternal and Postnatal Hla Diet on Circulating and Hepatic Lipids in Adult Offspring

**Figure 8:**
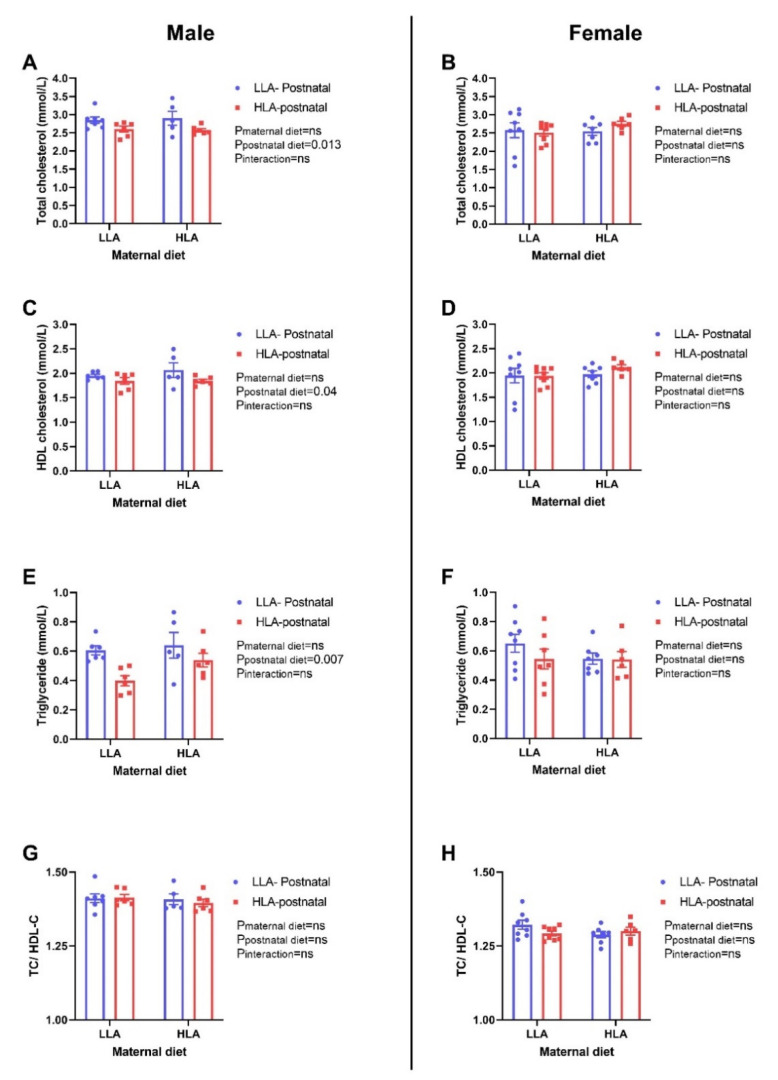
Effect of a maternal and postnatal diet high in linoleic acid on circulating lipid profile in adult offspring. Data are presented as mean ± standard error of the mean (SEM). Two-way ANOVA was performed for statistical analysis with maternal diet and postnatal diet as two factors. *n* = 5–8. LLA: low linoleic acid; HLA: high linoleic acid; PN: postnatal; TC: total cholesterol; HDL-C: high-density lipoprotein- cholesterol. **A**: Total cholesterol concentration male; **B**: Total cholesterol concentration female; **C**: HDL concentration male; **D**: HDL concentration female; **E**: Triglyceride concentration male; **F**: Triglyceride concentration female; **G**: Total cholesterol: HDL concentration male; **H**: Total cholesterol: HDL concentration female.

**Figure 9:**
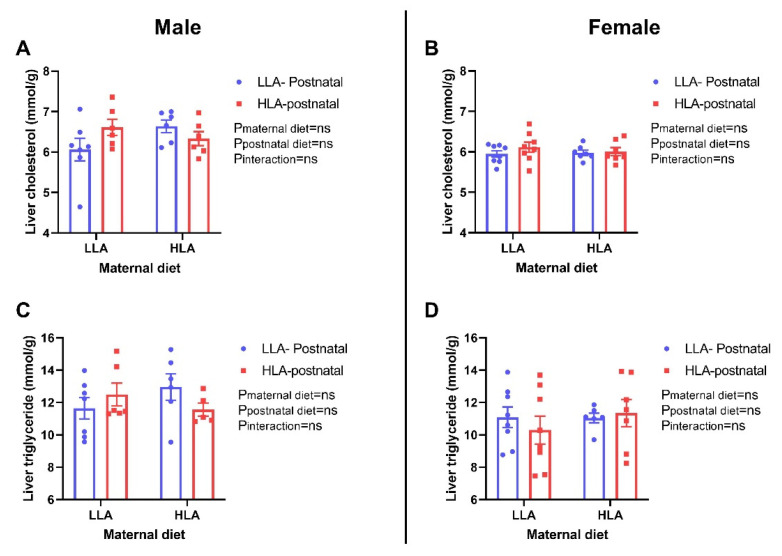
Effect of a maternal and postnatal diet high in linoleic acid on lipid concentration in the liver of adult offspring. Data are presented as mean ± standard error of the mean (SEM). Two-way ANOVA was performed for statistical analysis with maternal diet and postnatal diet as two factors. *n* = 6–8. LLA: low linoleic acid; HLA: high linoleic acid; PN: postnatal. **A**: Liver cholesterol concentration male; **B**: Liver cholesterol concentration female; **C**: Liver triglyceride concentration male; **D**: Liver triglyceride concentration female.

Postnatal HLA diet significantly lowered total cholesterol (*p* = 0.013), HDL-cholesterol (*p* = 0.04) and triglyceride (*p* = 0.007) in the plasma of male offspring, but not in female offspring (Figure 8). The ratio of total cholesterol and HDL-cholesterol was unchanged among the groups in both sexes. There were no significant differences in total cholesterol and triglyceride in liver of offspring in both sexes (Figure 9).

**Figure 10:**
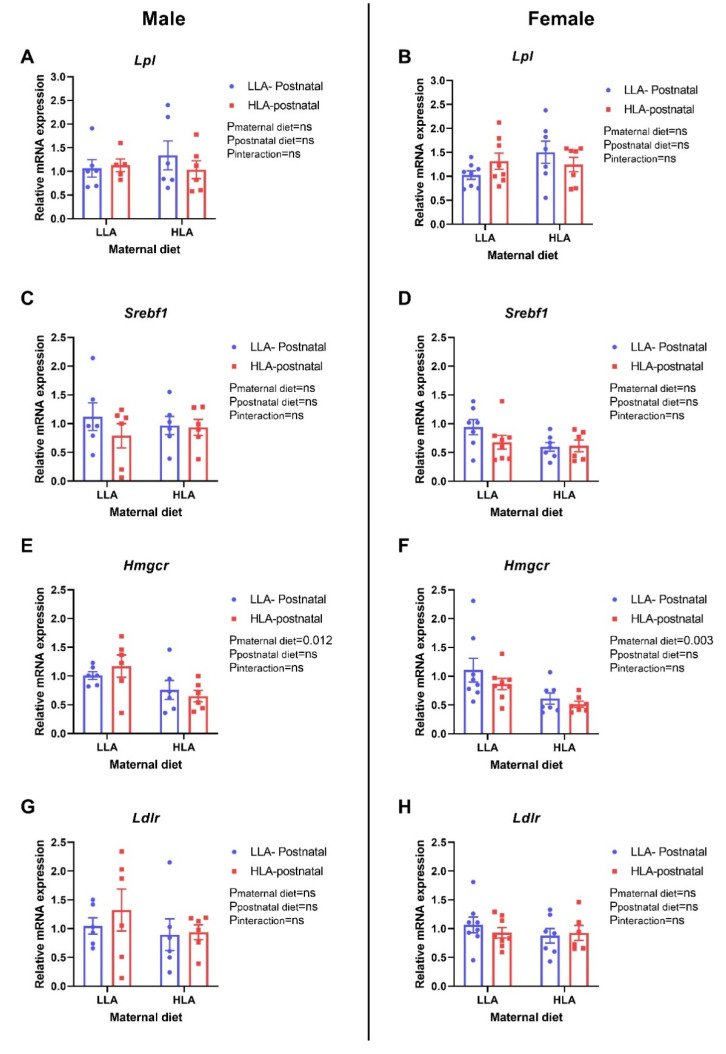
Effect of a maternal and postnatal diet high in linoleic acid on the expression of genes related to lipid metabolism in the liver of adult offspring. Data are presented as mean ± standard error of the mean (SEM). Two-way ANOVA was performed for statistical analysis with maternal diet and postnatal diet as two factors. *n* = 6–8. LLA: low linoleic acid; HLA: high linoleic acid; PN: postnatal. **A**: Lpl mRNA male; **B**: Lpl mRNA female; **C**: Srebf1 mRNA male; **D**: Srebf1 mRNA female; **E**: Hmgcr mRNA male; **F**: Hmgcr mRNA female; **G**: Ldlr mRNA male; **H**: Ldlr mRNA female.

**Figure 11:**
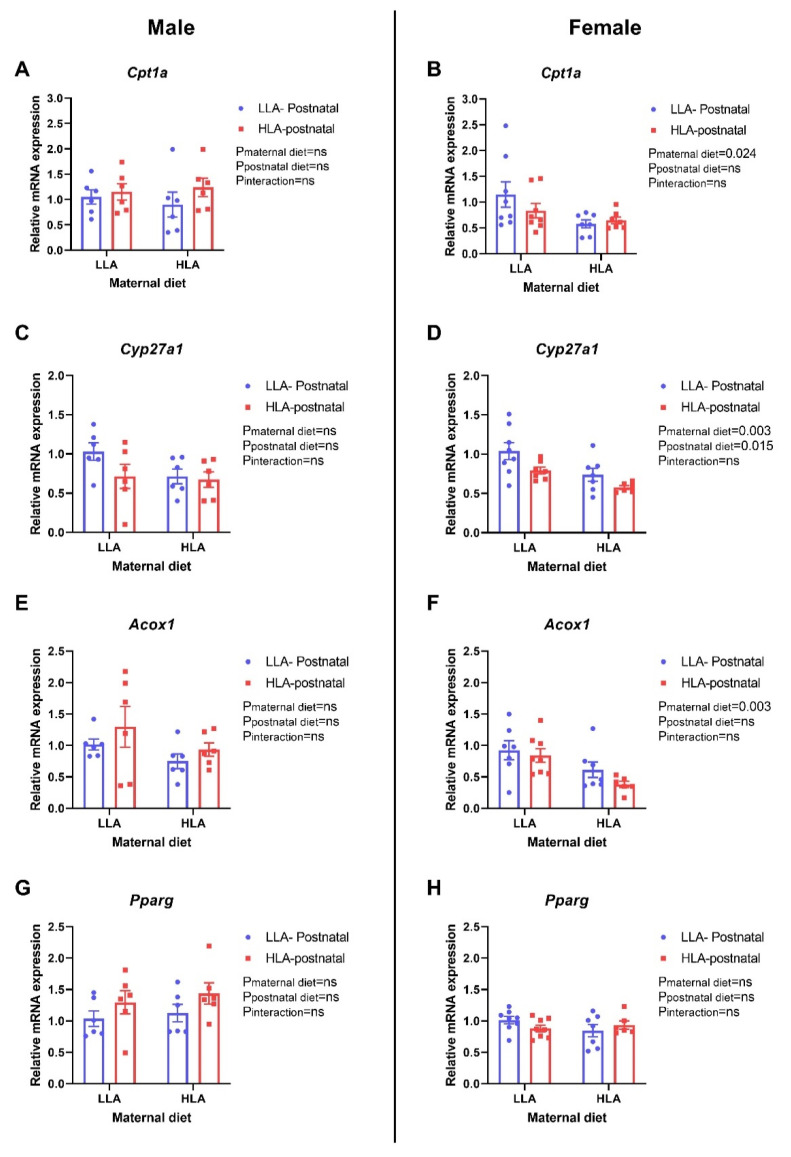
Effect of a maternal and postnatal diet high in linoleic acid on the expression of genes related to lipid metabolism in the liver of adult offspring. Data are presented as mean ± standard error of the mean (SEM). Two-way ANOVA was performed for statistical analysis with maternal diet and postnatal diet as two factors. *n* = 6–8. LLA: low linoleic acid; HLA: high linoleic acid; PN: postnatal. **A**: Cpt1a mRNA male; **B**: Cpt1a mRNA female; **C**: Cyp27a1 mRNA male; **D**: Cyp27a1 mRNA female; **E**: Acox1 mRNA male; **F**: Acox1 mRNA female; **G**: Pparg mRNA male; **H**: Pparg mRNA female.

There were no differences in the hepatic expression of Lpl and Srebf1 in the offspring of both sexes (Figure 10A–D). Maternal HLA diet significantly downregulated the relative gene expression of Hmgcr in both male (Figure 10E, *p* = 0.12) and female (Figure 10F, *p* = 0.003) offspring. There was no difference in the expression of Ldlr in the liver of either sexes. Maternal HLA diet significantly downregulated the expression of hepatic Cpt1a in female offspring (Figure 11B, *p* = 0.024), however, it remained unchanged in male offspring. Maternal (*p* = 0.003) and postnatal (*p* = 0.015) HLA diet significantly decreased hepatic expression of Cyp27a1 in female offspring (Figure 11D), however, there was no changes in the expression of Cyp27a1 in the liver of male offspring. The hepatic expression of Aocx1 was significantly downregulated by maternal HLA diet in female offspring (Figure 11F, *p* = 0.003), however, the expression of Acox1 remained unchanged in the liver of male offspring. There was no change in the expression of Pparg in the hepatic tissue of both sexes. 

## 3. Discussion

Chronic liver disease worldwide is increasing due to its association with metabolic syndrome [19]. A human study has previously shown that the n-6/n-3 FA ratio increases in non-alcoholic steatohepatitis, suggesting that increased LA in the diet may contribute to liver injury [19]. In the present study, we investigated the effect of a maternal diet high in LA on fatty acid composition, metabolic parameters and hepatic lipid metabolism in adult rat offspring. Further, we evaluated if the postnatal diet (LLA or HLA) reversed or exacerbated any adverse effect of the maternal diet. This study showed that hepatic lipid metabolism gene expression (*Hmgcr*, *Cpt1a*, *Cyp27a1*, *Acox1*), leptin and adiponectin are altered by maternal HLA diet, and plasma cholesterol and triglycerides are affected by postnatal HLA diet. 

The intake of LA in the human diet has been increasing in recent years [20], but the cardiac benefit of increased consumption of LA in the human diet is debated [21,22]. Several studies have shown that the substitution of SFA with PUFA lowers cholesterol and blood pressure, leading to a reduction in the incidence of heart disease [23,24]. However, the direct role of LA in metabolic health is still controversial [25]. The effect of LA on liver health is poorly understood, and only a few studies have investigated the impact of maternal LA directly on offspring fatty acid composition and hepatic lipid metabolism [26,27]. 

Studies have shown that maternal overnutrition increases the risk of offspring weight gain; [16,28] however, there are conflicting data as to whether the maternal dietary LA concentration contributes to this. A recent study has demonstrated that maternal HFD reduces offspring body weight independent of changes in the ratio of dietary LA/ALA [26]. Furthermore, in this study, maternal or postnatal diet did not affect offspring body weight in adulthood, which may be due to matched fat content in both LLA and HLA diets. We have also previously reported that an HLA diet before and during pregnancy does not alter the maternal body weight at gestation day 20 (E20) [29]. A previous study has also shown that an elevated LA diet does not affect body weight in mice [30]. In contrast, a study in a rat model showed that a PUFA diet rich in LA increased body weight compared to an SFA enriched diet, however, the n-3 PUFA was not matched in the experimental diet used in that study [31]. We did not observe a change in offspring body weight associated with an HLA diet.

Offspring plasma fatty acid concentrations may mirror the maternal intake of various fatty acids. We have recently demonstrated in a rat model that high maternal LA alters the fatty acid composition in both maternal and fetal circulations, and the placenta [29]. Furthermore, we have reported that maternal and postnatal HLA diets have a significant impact on plasma fatty acid composition in 40-day-old offspring (Hryciw et al., unpublished observation). In the present study, most of the fatty acids (MUFA, total n-3, ALA, DHA, total n-6, LA and ALA) in adult offspring were affected by postnatal diet but not by maternal diet. However, in adolescent offspring, most of these parameters were influenced by maternal diet as well. These findings suggest that maternal HLA diet has a greater role in adolescent offspring FA composition and postnatal HLA diet has more impact in FA composition in adult offspring. It is important to note that adolescent offspring were exposed to a postnatal diet for only 15 days, while adult offspring were exposed to a postnatal diet for 5 months, and therefore the stronger effect of a postnatal diet in adult offspring may be an effect of a longer period of exposure. Despite the matched SFA content in the diets used in the current investigations, the postnatal HLA diet significantly decreased total SFA in the plasma of adult male offspring, with no difference in female offspring. 

Fat mass is an important indicator of metabolic health [32,33] and an increase in fat mass is associated with an increased risk of developing liver disease [34]. The findings from a randomized controlled trial have shown that higher erythrocyte LA is associated with decreased trunk adipose mass and increased appendicular lean mass [35]. In mice, reduced LA intake in the early postnatal period has been shown to lower fat mass in adulthood following a Western-style diet challenge [36]. In the present study, maternal or postnatal HLA diet did not affect the fat mass and lean mass in adult offspring. Leptin, an adipokine hormone secreted by adipose tissue, is associated with body weight and fat mass [37]. Even though there were no changes in body weight and fat mass, a maternal HLA diet increased circulating leptin concentrations in adult female offspring. We have previously shown that in pregnant rats consuming an HLA diet, their circulating leptin is reduced [29]. It is important to note that leptin is also associated with reproductive physiology, [38,39] ageing [40] and immune function, [41] which were not assessed in this study. In addition, leptin plays a major role in liver lipid handling, as recombinant leptin reverses insulin resistance and hepatic steatosis in lipodystrophic patients [42]. Adiponectin, another adipokine, is a classic anti-inflammatory agent and also possesses anti-fibrotic properties [43]. Adiponectin is known to increase insulin sensitivity, with low adiponectin plasma concentrations associated with insulin resistance [43]. In our study, a maternal HLA diet decreased plasma adiponectin concentrations in male offspring, however, there was no change in insulin sensitivity as shown by the insulin tolerance test. Similarly, a maternal or postnatal HLA diet did not affect glucose tolerance in the adult offspring. A previous study has shown that LA decreases adiponectin secretion from primary rat adipocytes in the presence of insulin, however, LA had no effect on basal adiponectin secretion [44]. The finding from the Munich LISAplus birth cohort study, demonstrated that a higher n-3 PUFA and a lower n-6/n-3 PUFA ratio in cord blood are associated with increased adiponectin at 10 years of age, [45] suggesting that LA in plasma maybe associated with circulating adiponectin. Alteration of adiponectin concentrations is associated with change in insulin sensitivity, regulation of food intake and energy expenditure [43]. This insulin sensitizing adipokine also has anti-inflammatory, anti-fibrotic and proangiogenic effects [43]. However, there may be effect on offspring cardiac function which was not evaluated in the current study. Leptin and adiponectin regulate lipid metabolism as well, so we were interested to observe the role of linoleic acid on hepatic lipid metabolism. 

Several lines of evidence have reported that increased SFA contributes to the development of metabolic diseases including liver disease [46,47]. Interestingly, in this study, a postnatal HLA diet decreased total n-3 and ALA in the plasma of both male and female offspring irrespective of maternal diet, while DPA in male offspring and EPA in female offspring were decreased by both maternal and postnatal HLA diet. Recently, a study showed that a high maternal LA diet decreased whole blood total n-3 fatty acid in 8-week-old offspring of both sexes, despite the offspring no longer being directly treated with HLA diet [26]. A meta-analysis has shown that higher circulating n-3 PUFA are associated with a lower metabolic syndrome risk [48] suggesting that lower n-3 PUFA may increase risk of disease. However, in the present study, we have not observed a change in the markers such as blood glucose, fat mass and blood pressure. 

As expected, total n-6 PUFA ad LA were significantly elevated by a postnatal HLA diet in both male and female offspring. Interestingly, AA, an inflammatory metabolite of LA, was also increased in the plasma of male and female offspring exposed with HLA diet postnatally. Furthermore, a postnatal HLA diet elevated the plasma AA/DHA ratio in both male and female offspring in the current study. A previous in-vitro study has shown that a high AA/DHA ratio promotes intracellular triglyceride accumulation and decreases mitochondrial activity in human hepatoma cells [49]. Elevated AA/DHA ratio in plasma of offspring exposed to HLA diet postnatally suggests that this may impact lipid metabolism in liver. 

Serum liver enzymes, namely ALT and AST, are regarded as liver injury markers, as well as strong predictors of metabolic disease [50]. In this study, a postnatal HLA diet lowered plasma AST in female offspring, however, ALT and ALP remained unchanged among the group in both male and female offspring. Circulating liver enzymes are used in clinical practice to monitor the progression of liver injury [51] and alteration in liver enzymes is often associated with disturbances in lipid metabolism in the liver [52]. Hence, the change in AST in our study may contribute to alterations in lipid metabolism in the liver. Furthermore, elevated plasma uric acid are associated with dyslipidaemia and play a vital role in the development of metabolic syndrome [53]. In our present study, a postnatal HLA diet elevated plasma uric acid in adult female offspring, which may be associated with change in lipids in circulation. Furthermore, uric acid is excreted by the proximal tubules and is a marker for kidney disease and gout [54]. 

Systematic assessment of randomized controlled trials has shown that increased n-6 PUFA and LA lower serum total cholesterol [55]. We have previously reported that LA treatment before and during pregnancy lowers total cholesterol, LDL-cholesterol and HDL-cholesterol in maternal circulation in a rat model [29]. In addition, we observed a decrease in total cholesterol and HDL-cholesterol in adolescent offspring from the dams fed with HLA. However, in the present study, a postnatal HLA diet lowered total cholesterol, HDL-cholesterol and triglyceride in adult male offspring only. It is interesting that a maternal HLA diet lowered total cholesterol in female offspring during adolescence while, in contrast, a postnatal HLA diet lowered total cholesterol in male offspring during adulthood. There was no change in triglycerides in maternal or adolescent offspring circulation due to HLA diet, however, it has been impacted in adult male offspring. In contrast to our animal study, a human study in a Mexican population showed that a higher dietary n-6/n-3 PUFA ratio is associated with higher blood triglyceride, [56] and in another rat model, a high LA diet (n-6/n-3 ratio = 11.4/1) did not later plasma lipids [57]. These studies highlight the controversies in the lipid lowering effect of n-6 PUFA or LA. 

We further investigated the effect of a maternal or postnatal HLA diet on lipids and expression of genes related to lipid metabolism in the liver of adult offspring. A previous study in a rat model has shown that exposure to a high LA (36% fat) diet during pregnancy results in reduced liver triglyceride in 8-week-old male offspring but not in female offspring [26] While there are multiple randomized controlled trials that studied effects of LA on plasma cholesterol, [55] there is a paucity of data showing effect on liver lipids, probably due to lack of feasibility in humans. In our study, a maternal or postnatal HLA diet did not affect cholesterol or triglyceride in the liver of adult offspring. A previous study has shown that 5% LA in presence of 0.1% dietary cholesterol lowers LDL-cholesterol in the plasma of male Golden Syrian hamsters, however, it did not affect hepatic cholesterol content [58]. This study further demonstrated that LA in presence of cholesterol suppresses HMG-CoA reductase (HMGCR) activity [59]. In agreement, a maternal HLA diet lowered the expression of hepatic *Hmgcr* in both male and female offspring in our current study, without alteration in hepatic lipid concentrations. HMGCR is a rate-limiting enzyme in cholesterol synthesis [58], therefore lower expression may lead to reduced cholesterol synthesis. A diet with a lower ratio of n-6/EPA + DHA (20:1) decreased the expression of *Hmgcr* in liver and decreased hepatic lipid accumulation in mice [60]. Furthermore, we were interested to observe the effect of maternal or postnatal HLA diet on the gene involved in beta-oxidation, as mitochondrial oxidation of FA plays a vital role in development of lipid dysfunction in liver. Carnitine palmitoyl-transferase 1 (CPT1) is a rate-limiting enzyme of beta-oxidation that facilitates import of fatty acid through the outer mitochondrial membrane [61]. *Cpt1a*, a liver isoform of CPT1, was significantly downregulated by maternal HLA diet in female offspring in our current study. *Cpt1a* is known to be affected by dietary interventions and environmental factors through various molecular mechanisms. The findings from the Dutch Famine cohort has shown the positive correlation between prenatal malnutrition and methylation within a *Cpt1a* enhancer [62]. In a rat model, HFD exposure from gestation through early adulthood induced lipid accumulation and increased *Cpt1a* expression in liver of 12-week-old male offspring [63]. However, in our present study, *Cpt1a* expression changed without alteration in the hepatic lipid content. Acyl-CoA oxidase 1 (Acox1) is essential for peroxisomal beta- oxidation and hydrogen peroxide homeostasis in the liver and dysregulation of Acox1 can lead to the development of chronic liver disease [64]. In our current study, a maternal HLA diet decreased hepatic *Acox1* gene expression in female offspring. Downregulation of the lipogenic gene (*Hmgcr*) as well as genes involved in beta-oxidation (*Cpt1a, Acox1*), which may suggest the controversial role of HLA diet in lipid metabolism in the liver. Furthermore, sterol 27-hydroxylase (CYP27A1) is an enzyme required for bile acid synthesis from cholesterol and involved in cholesterol degradation in liver [65]. Interestingly, hepatic expression of *Cyp27a1* was downregulated by a maternal as well as a postnatal HLA diet in female offspring. In human hepatic cell model, n-3 PUFA reduced the expression of *Cyp27a1*, however, the role of maternal PUFA in offspring bile acid synthesis has not been studied. Our study suggests that maternal LA may affect the bile acid synthesis in offspring. A future direction for this project is to confirm changes in mRNA, reflect functional protein and enzyme expression.

In conclusion, a maternal or postnatal diet high in LA alters fatty acid composition, leptin and adiponectin in the plasma of adult offspring. Maternal or postnatal HLA diet did not affect physiological metabolic parameters such as systolic blood pressure, fat mass, glucose tolerance and insulin sensitivity. Furthermore, a postnatal HLA diet decreased total cholesterol, HDL-cholesterol and triglyceride in the male offspring but not in female offspring. Interestingly, a maternal HLA diet alters the expression of genes related to lipid metabolism in the liver but not hepatic lipid content. These findings suggest that maternal or postnatal diet high in LA are independent factors for offspring metabolic parameters and genes involved in hepatic lipid metabolism. Sex specific difference observed in the current study might be the effect of sex hormones, however, it is not investigated in this study. Further studies should be conducted to identify the exact mechanism of altered lipid metabolism in offspring liver due to maternal HLA diet. 

## 4. Materials and Methods

### 4.1. Experimental Animal Model and Diet

Ethical approval was granted by the Griffith University Animal Ethics Committee (NSC/01/17/AEC: 26 April 2017). Wistar Kyoto rats (8 weeks of age; *n* = 8 for diet with low linoleic acid (LLA) and *n* = 10 for diet with high linoleic acid (HLA)) were purchased from the Australian Resource Centre (ARC, Kensington, WA, Australia) and housed in accordance to the Australian Code of Practice for Care and Use of Animals for Scientific Purpose, following the ARRIVE Guidelines for Reporting Animal Research. 

Eight-week-old female Wistar Kyoto (WKY) rats were housed in individually ventilated cages under 12 h light-dark cycle at a temperature of 20–22 °C and provided with standard food pellets during acclimatisation and tap water ad libitum throughout the study. After a week for acclimatization, female rats were randomised to consume either a control low LA (LLA: 1.44% of energy from LA, *n* = 8) or high LA (HLA: 6.21% of energy from LA, *n* = 8) diet for 10 weeks. The minimum requirement for LA in the rodent diet is between 1–1.5% [66]. The composition of the custom diet has been previously reported [29]. These diets were isocaloric and matched for n-3 PUFA and total fat content. Offspring from mothers were weaned at PN25 and fed with either a LLA or HLA diet. Systolic blood pressure in the adult offspring was measured at PN160 (as described below). An intraperitoneal glucose tolerance test (GTT) was performed in offspring at PN166 (as described below). An intraperitoneal insulin tolerance test (ITT) was performed at PN170 (as described below). Body composition of adult offspring was evaluated using DXA (as described below). Offspring were terminally anesthetised with an intraperitoneal injection of sodium pentobarbital (60 mg/kg) and scanned under DXA before cardiac puncture. Organs were weighed and fixed for histology or frozen for gene analysis. Blood samples were collected by cardiac puncture, centrifuged at 5000× *g* for 10 min to separate plasma and stored at −80 °C for analysis.

### 4.2. Physiological Experiments in Adult Offspring

#### 4.2.1. Non-Invasive Measurement of Systolic Blood Pressure in Pn180 Offspring

Systolic blood pressure (SBP) of offspring was measured using non-invasive blood pressure system (NIBP) system (AD Instruments, Sydney, NSW, Australia) at PN160. It includes a tail cuff and pulse transducer that measure blood pressure based on the periodic occlusion of tail blood flow. The NIBP system function with PowerLab data acquisition units and SBP is monitored in LabChart software (AD Instruments). Rats were warmed for 5 min and restrained using rat restrainer (AD Instruments). The inflatable tail cuff was placed around the proximal end of the rat tail and the pulse transducer was placed adjacent to the cuff. The cuff was inflated, and the blood pressure was recorded using LabChart software. Rats were habituated for the restrainer and cuff inflation before actual blood pressure measurement.

#### 4.2.2. Intraperitoneal Glucose Tolerance Test in Pn180 Offspring

The glucose tolerance of the rat offspring was assessed by an intraperitoneal glucose tolerance test (IPGTT) at PN166. Rats were transferred into new clean cage and fasted for 16 hr. The first blood samples were collected from tail tip and blood glucose was measured using a glucometer which was considered as time 0 min. Subsequently, a 50% glucose solution (1 gm/kg of body weight) was administrated intraperitoneally into the rats and blood glucose was monitored at 15, 30, 60, 90 and 120 min after glucose administration. The response to glucose was calculated as area under the curve (AUC) for individual rat and analysed using GraphPad Prism software 8.3.1 (GraphPad Software, San Diego, CA, USA).

#### 4.2.3. Intraperitoneal Insulin Tolerance Test in Adult Offspring

The insulin tolerance of the rat offspring was assessed by an intraperitoneal insulin tolerance test (IPITT) at PN170. After 2 h of fasting, blood samples were collected from the rat’s tail tip and blood glucose was measured using a glucometer which was considered as time 0 min. Subsequently, rats were intraperitoneally injected with 0.75 U/kg body weight of recombinant human insulin (Novo Nordisk, Bagsværd, Denmark). Blood glucose was monitored at 15, 30, 60, 90 and 120 min after insulin administration. The response to insulin was calculated as area under the curve (AUC) for individual rats and analysed using GraphPad Prism software version 8.3.1.

#### 4.2.4. Body Composition Measurement in Adult Offspring

Fat mass and lean mass in PN180 rat offspring were measured using DXA. Rats were transported to the DXA lab from the animal housing facility on the day of PN180. Rats were anesthetized with an intraperitoneal injection of sodium pentobarbital (60 mg/kg). Anaesthetized and completely immobile rats were placed on this clear plastic in the middle of scanner bed and scanned under DXA.

#### 4.2.5. Fatty Acid Analysis in Offspring Plasma

The concentrations of fatty acids in maternal and fetal plasma were measured using Gas chromatography (GC) as previously described [67]. Briefly, 50 μL of plasma sample was spotted onto blood collection paper and dried in air at room temperature. The samples were analysed by the South Australian Health and Medical Research Institute, following the method described in [67]. The fatty acid content was expressed as total lipid fatty acids as %.

#### 4.2.6. Measurement of Circulating Leptin Concentration

Leptin concentration in maternal and offspring plasma was estimated by ELISA following the manufacture’s guidelines (Mouse/Rat Leptin Quantikine ELISA Kit, R&D Systems, Minneapolis, MN, USA). The intra-assay coefficient of variation was <3.0%. 

#### 4.2.7. Biochemical Analysis

Plasma biochemical parameters were assessed using an automated chemistry analyser (Integra 400 plus, Roche Diagnostics, North Ryde, NSW, Australia) in EDTA anticoagulated plasma. All biochemistry assays were performed using Roche certified assay kits, which were calibrated using Calibrator for Automated Systems reagent. Quality control standards (PreciControl ClinChem Multi 1 and 2; Roche Diagnostics) were run prior to sample analysis to ensure accuracy of results. All analyses were performed in duplicate.

#### 4.2.8. Plasma Adiponectin Estimation in Adult Offspring

Adiponectin concentration in the plasma of adult offspring was measured using a commercial ELISA kit following the manufacture’s guidelines (Abcam, Cambridge, UK). The intra-assay coefficient of variation was <3.0%.

#### 4.2.9. Cholesterol and Triglyceride Quantification in the Liver of Offspring

Lipids from the liver tissue were extracted following previously published methods [68]. Liver tissue was homogenised while frozen (liquid nitrogen) using a mortar and pestle and the weight was determined. Lipids from the homogenized sample were extracted by two sequential solvent extractions with isopropanol. During each extraction, homogenates were vortexed, sonicated for 10 min and then centrifuged at 43,000× *g* for 10 min. The two supernatant fractions were collected in new 15 mL falcon tubes and evaporated using the Roto—evaporator (Maxivac, Labogene, Bjarkesvej Lillerød, Denmark) at 35 °C (500 RPM, 1kPa pressure). The remaining dry pellet was reconstituted in 250 µL of isopropanol and loaded into an automated biochemistry analyser (COBAS Integra 400+, Roche Diagnostics) for the quantification of total cholesterol and triglyceride. Total cholesterol and triglyceride kits were purchased from Roche Diagnostics. These kits were verified with their appropriate calibrators and quality control (QC) prior to sample analysis. Isopropanol did not interfere cholesterol or triglyceride contents.

#### 4.2.10. Quantitative Real Time Polymerase Chain Reaction (qPCR)

Total RNA was extracted from liver tissue using RNeasy Mini kit (Qiagen, Chadstone, VIC, Australia) following the manufacturer’s guidelines. The quantification and evaluation of purity of RNA samples was assessed using the NanoDrop 1000 spectrophotometer (Thermo Fisher Scientific, Waltham, MA, USA). Reverse transcription of RNA to synthesize complementary DNA was performed using the iScript gDNA clear cDNA synthesis kit (BioRad, Hercules, CA, USA) following manufacturer’s guidelines. Quantitative PCR was performed using QuantiNova SYBR^®^ green master mix (Qiagen) following manufacturer’s guidelines, in line with the Minimum Information for Publication of Quantitative Real-Time PCR Experiments (MIQE) guidelines [69]. PCR initial heat activation was run for 2 min at 95 °C, then qPCR reactions were run for 40 cycles of 95 °C for 5 s (denaturation) and 60 °C for 10 s (combined annealing/extension) using StepOne^TM^ real-time PCR systems (Applied Biosystems, Waltham, MA, USA). Gene expression was quantified using the 2^-ΔΔ*C*q^ method normalised to the geometric mean of β-actin and β-2 microglobulin as reference genes. These reference genes were stable across the treatment groups.

#### 4.2.11. Statistical Analysis

All data were analysed using GraphPad Prism 8.3.1. One male and one female offspring from each litter were analysed. n values represent individual offspring from separate litters. Data were analysed separately for males and female with each sex analysed by two-way ANOVA, with maternal and postnatal diet as the factors. Specific comparisons were made using Tukey post-hoc test. Data are presented as mean ± standard error of the mean (SEM). *p*-values < 0.05 were considered evidence of significant differences.

## Figures and Tables

**Table 1:** Effect of a maternal and postnatal diet high in linoleic acid on body weight and organ weight of adult offspring.

Organ and Body Weight	LLA Maternal Diet		HLA Maternal Diet		Two-Way ANOVA		
	LLA PN Diet	HLA PN Diet	LLA PN Diet	HLA PN Diet	Pmaternal	Ppostnatal	Pint
Male							
Body weight (gm)	388.2 ± 5.4600	378.3 ± 6.5800	384.2 ± 6.3900	374.9 ± 5.0900	ns	ns	ns
% Left kidney	0.314 ± 0.0050	0.313 ± 0.0010	0.316 ± 0.0030	0.310 ± 0.0050	ns	ns	ns
% Right kidney	0.317 ± 0.0060	0.307 ± 0.0020	0.309 ± 0.0040	0.307 ± 0.0060	ns	ns	ns
% Left adrenal	0.005 ± 0.0001	0.005 ± 0.0005	0.004 ± 0.0001	0.004 ± 0.0002	ns	ns	ns
% Right adrenal	0.004 ± 0.0002	0.004 ± 0.0001	0.004 ± 0.0005	0.004 ± 0.0003	ns	ns	ns
% Liver	3.06 ± 0.0300	2.95 ± 0.0300	2.97 ± 0.0500	2.95 ± 0.0500	ns	ns	ns
% Heart	0.333 ± 0.0100	0.337 ± 0.0100	0.331 ± 0.0060	0.327 ± 0.0020	ns	ns	ns
% Brain	0.541 ± 0.0090	0.571 ± 0.0050	0.555 ± 0.0080	0.576 ± 0.0050	ns	*p* = 0.003	ns
% GAS	0.554 ± 0.0100	0.548 ± 0.0090	0.558 ± 0.0100	0.580 ± 0.0200	ns	ns	ns
% SOL	0.035 ± 0.0010	0.036 ± 0.0009	0.035 ± 0.0010	0.036 ± 0.0010	ns	ns	ns
% TA	0.163 ± 0.0060	0.159 ± 0.0010	0.159 ± 0.0060	0.160 ± 0.0020	ns	ns	ns
% EDL	0.047 ± 0.0020	0.051 ± 0.0007	0.050 ± 0.0006	0.047 ± 0.0020	ns	ns	ns
Female							
Body weight (gm)	230.6 ± 4.0000	240.2 ± 2.2800	241.4 ± 2.0200	238.1 ± 3.9400	ns	ns	ns
% Left kidney	0.304 ± 0.0040	0.313 ± 0.0010	0.305 ± 0.0040	0.296 ± 0.0040	ns	ns	*p* = 0.03
% Right kidney	0.306 ± 0.0070	0.316 ± 0.0030	0.302 ± 0.0010	0.305 ± 0.0060	ns	ns	ns
% Left adrenal	0.01 ± 0.0010	0.01 ± 0.0006	0.01 ± 0.0005	0.01 ± 0.00010	ns	ns	ns
% Right adrenal	0.01 ± 0.0009	0.01 ± 0.0003	0.01 ± 0.0007	0.01 ± 0.00090	ns	ns	ns
% Liver	3.69 ± 0.0500	3.68 ± 0.0200	3.67 ± 0.0500	3.61 ± 0.0100	ns	ns	ns
% Heart	0.346 ± 0.0050	0.384 ± 0.0200	0.357 ± 0.0040	0.352 ± 0.0050	ns	ns	ns
% Brain	0.847 ± 0.0120	0.825 ± 0.0140	0.835 ± 0.0090	0.844 ± 0.0110	ns	ns	ns
% GAS	0.604 ± 0.0200	0.583 ± 0.0100	0.594 ± 0.0100	0.575 ± 0.010	ns	ns	ns
% SOL	0.042 ± 0.0010	0.037 ± 0.0010	0.041 ± 0.0008	0.043 ± 0.0010	ns	ns	*p* = 0.011
% TA	0.174 ± 0.0020	0.172 ± 0.0020	0.165 ± 0.0010	0.164 ± 0.0040	*p* = 0.005	ns	ns
% EDL	0.050 ± 0.0010	0.050 ± 0.0009	0.055 ± 0.0010	0.055 ± 0.0010	*p* = 0.002	ns	ns
% Left ovary	0.015 ± 0.0008	0.016 ± 0.0010	0.017 ± 0.0008	0.017 ± 0.0010	ns	ns	ns
% Right ovary	0.017 ± 0.0008	0.016 ± 0.0008	0.017 ± 0.0008	0.018 ± 0.0005	ns	ns	ns

Data are presented as mean ± standard error of the mean (SEM). Two-way ANOVA was performed for statistical analysis with maternal diet and postnatal diet as two factors. *n* = 6–8. LLA: low linoleic acid; HLA: high linoleic acid; PN: postnatal; GAS: gastrocnemius; SOL: soleus; TA: tibialis anterior; EDL: extensor digitorum longus. ns: not significant

**Table 2:** Effect of a maternal and postnatal diet high in linoleic acid on plasma fatty acid composition in adult offspring.

Plasma Fatty Acid	LLA Maternal Diet		HLA Maternal Diet		Two-Way ANOVA		
	LLA PN Diet	HLA PN Diet	LLA PN Diet	HLA PN Diet	Pmaternal	Ppostnatal	Pint
Male							
Total SFA	33.41 ± 0.3600	32.51 ± 0.2100	33.85 ± 0.1800	32.61 ± 0.3300	ns	*p* = 0.003	ns
Total trans FA	0.10 ± 0.0000	0.10 ± 0.0000	0.10 ± 0.0000	0.10 ± 0.0000	ns	ns	ns
Total MUFA	15.61 ± 1.4300	7.83 ± 0.3100	14.56 ± 0.9700	9.11 ± 0.5400	ns	*p* < 0.0001	ns
Total n-9 FA	12.27 ± 1.1500	5.70 ± 0.2300	11.12 ± 0.8100	6.58 ± 0.3900	ns	*p* < 0.0001	ns
Total n-7 FA	3.28 ± 0.3300	2.08 ± 0.1100	3.34 ± 0.3300	2.55 ± 0.1600	ns	*p* = 0.0011	ns
Total n-3 FA	3.65 ± 0.1200	2.63 ± 0.0600	3.80 ± 0.0800	2.66 ± 0.0800	ns	*p* < 0.0001	ns
ALA (18:3n-3)	0.32 ± 0.0400	0.21 ± 0.0100	0.26 ± 0.0400	0.20 ± 0.0000	ns	*p* = 0.012	ns
EPA (20:5n-3)	0.25 ± 0.0600	0.10 ± 0.0000	0.20 ± 0.0000	0.10 ± 0.0000	ns	*p* = 0.04	ns
DPA (22:5n-3)	0.44 ± 0.0200	0.35 ± 0.0200	0.50 ± 0.0300	0.40 ± 0.0000	*p* = 0.019	*p* = 0.0002	ns
DHA (22:6n-3)	2.64 ± 0.0600	2.03 ± 0.0800	2.86 ± 0.1100	2.01 ± 0.0700	ns	*p* < 0.0001	ns
Total n-6 FA	47.21 ± 1.7400	56.93 ± 0.300	48.20 ± 0.8800	55.50 ± 0.6900	ns	*p* < 0.0001	ns
LA (18:2n-6)	16.44 ± 0.6100	20.60 ± 0.5500	15.74 ± 1.0600	20.45 ± 0.6400	ns	*p* < 0.0001	ns
AA (20:4n-6)	29.24 ± 1.0700	34.18 ± 0.5600	30.92 ± 0.6800	32.76 ± 0.7100	ns	*p* = 0.0006	ns
LA/ALA	56.47 ± 9.1500	105.8 ± 5.9000	58.47 ± 4.5100	99.92 ± 3.6000	ns	*p* < 0.0001	ns
AA/DHA	11.1 ± 0.5900	16.81 ± 0.5600	10.85 ± 0.2800	16.31 ± 0.3900	ns	*p* < 0.0001	ns
DHA/DPA	5.96 ± 0.3100	6.02 ± 0.3200	5.98 ± 0.3800	5.37 ± 0.1100	ns	ns	ns
Female							
Total SFA	34.55 ± 0.3100	34.90 ± 0.2500	35.02 ± 0.3800	35.02 ± 0.1900	ns	ns	ns
Total trans FA	0.12 ± 0.0100	0.10 ± 0.0000	0.10 ± 0.0000	0.10 ± 0.0000	ns	ns	ns
Total MUFA	13.74 ± 0.7800	8.82 ± 0.5600	12.45 ± 0.7300	7.81 ± 0.3700	ns	*p* < 0.0001	ns
Total n-9 FA	11.20 ± 0.6900	6.63 ± 0.4700	9.94 ± 0.6400	5.75 ± 0.2900	ns	*p* < 0.0001	ns
Total n-7 FA	2.45 ± 0.0900	2.13 ± 0.1000	2.40 ± 0.0800	1.95 ± 0.0900	ns	*p* = 0.0005	ns
Total n-3 FA	4.94 ± 0.1500	3.86 ± 0.0500	4.92 ± 0.1500	3.96 ± 0.0500	ns	*p* < 0.0001	ns
ALA (18:3n-3)	0.28 ± 0.0400	0.14 ± 0.0100	0.20 ± 0.0000	0.15 ± 0.0200	ns	*p* = 0.0009	ns
EPA (20:5n-3)	0.15 ± 0.0300	0.10 ± 0.0000	0.10 ± 0.0000	0.04 ± 0.0200	*p* = 0.014	*p* = 0.014	ns
DPA (22:5n-3)	0.27 ± 0.0200	0.21 ± 0.0100	0.20 ± 0.0200	0.20 ± 0.0000	*p* = 0.04	ns	ns
DHA (22:6n-3)	4.22 ± 0.1300	3.48 ± 0.0600	4.41 ± 0.1400	3.56 ± 0.0600	ns	*p* < 0.0001	ns
Total n-6 FA	46.64 ± 0.7700	52.27 ± 0.6200	47.48 ± 0.6600	53.40 ± 0.4400	ns	*p* < 0.0001	ns
LA (18:2n-6)	14.20 ± 0.9800	16.02 ± 0.8100	13.51 ± 0.9000	16.98 ± 0.7800	ns	*p* = 0.006	ns
AA (20:4n-6)	30.94 ± 0.8500	34.01 ± 0.8300	32.38 ± 0.4400	34.25 ± 0.6000	ns	*p* = 0.002	ns
LA/ALA	52.21 ± 4.8400	111.01 ± 5.3400	79.22 ± 6.2600	118.23 ± 2.8600	*p* = 0.004	*p* < 0.0001	ns
AA/DHA	7.34 ± 0.1400	9.77 ± 0.2400	7.34 ± 0.1500	9.31 ± 0.2300	ns	*p* < 0.0001	ns
DHA/DPA	16.87 ± 1.0800	17.27 ± 1.1100	20.65 ± 1.0100	18.39 ± 0.6700	*p* = 0.02	ns	ns

Data are presented as mean ± standard error of the mean (SEM). Two-way ANOVA was performed for statistical analysis with maternal diet and postnatal diet as two factors. *n* = 5–8. LLA: low linoleic acid; HLA: high linoleic acid; PN: postnatal; FA: fatty acid; SFA: saturated fatty acid; MUFA: monosaturated fatty acid; ALA: α- linolenic acid; EPA: eicosapentaenoic acid; DPA: docosapentaenoic acid; DHA: docosahexaenoic acid; LA: linoleic acid; AA: arachidonic acid.

## Supplementary Materials

The following are available online at https://www.mdpi.com/1422-0067/22/6/2946/s1
