# Supplementary material for: Maternal and Postnatal High Linoleic Acid Diet Impacts Lipid Metabolism in Adult Rat Offspring in a Sex-Specific Manner
Source: Int J Mol Sci. 2021 Mar 14;22(6):2946. doi: 10.3390/ijms22062946 (PMC7999727; doi:10.3390/ijms22062946)
Supplement: Supplementary file 1 [file ijms-22-02946-s001.pdf]

Figure 1. Absolute organ weight.

Organ Weight	LLA Maternal Diet		HLA Maternal Diet		Two-Way ANOVA		
	**LLA PN Diet**	**HLA PN Diet**	**LLA PN Diet**	**HLA PN Diet**	**Pmaternal**	**Ppostnatal**	**Pint**
**Male**							
Left kidney	1.22 ±0.0330	1.19 ± 0.0220	1.20 ± 0.0210	1.16 ± 0.0210	ns	ns	ns
Right kidney	1.23 ± 0.0330	0.16 ± 0.0280	1.18 ± 0.0220	1.15 ± 0.0240	ns	ns	ns
Left adrenal	0.02 ± 0.0008	0.02 ± 0.0020	0.02 ± 0.0004	0.02 ± 0.0010	ns	ns	ns
Right adrenal	0.02 ± 0.0010	0.02 ± 0.0010	0.02 ± 0.0020	0.02 ± 0.0010	ns	ns	ns
L. Kidney + L. Adrenal	1.24 ± 0.0330	1.21 ± 0.020.	1.22 ± 0.0220	1.18 ± 0.0210	ns	ns	ns
R. Kidney + R. Adrenal	1.25 ± 0.0330	1.18 ± 0.0290	1.20 ± 0.0210	1.17 ± 0.0230	ns	ns	ns
Liver	11.9 ± 0.2810	11.2 ± 0.270	11.4 ± 0.1840	11.1 ± 0.1710	ns	0.03	ns
Heart	1.29 ± 0.0580	1.27 ± 0.0500	1.27 ± 0.0230	1.23 ± 0.0170	ns	ns	ns
Brain	2.09 ± 0.019	2.16 ± 0.0240	2.13 ± 0.0220	2.16 ± 0.0130	ns	0.03	ns
GAS	2.10 ± 0.0240	2.12 ± 0.0290	2.14 ± 0.0480	2.17 ± 0.0540	ns	ns	ns
SOL	0.14 ± 0.0050	0.14 ± 0.0040	0.14 ± 0.0050	0.14 ± 0.0050	ns	ns	ns
TA	0.64 ± 0.0250	0.59 ± 0.0200	0.61 ± 0.0190	0.62 ± 0.0200	ns	ns	ns
EDL	0.19 ± 0.0050	0.20 ± 0.0050	0.19 ± 0.0040	0.19 ± 0.0070	ns	ns	ns
**Female**							
Left kidney	0.70 ± 0.0160	0.74 ± 0.0070	0.73 ± 0.0090	0.71 ± 0.0220	ns	ns	0.02
Right kidney	0.70 ± 0.0170	0.76 ± 0.0070	0.73 ± 0.0080	0.73 ± 0.0150	ns	ns	0.04
Left adrenal	0.03 ± 0.0040	0.03 ± 0.0020	0.03 ± 0.0010	0.03 ± 0.0020	ns	ns	ns
Right adrenal	0.02 ± 0.0020	0.03 ± 0.0020	0.03 ± 0.0020	0.03 ± 0.0020	ns	ns	ns
L. Kidney + L. Adrenal	0.73 ± 0.0180	0.77 ± 0.0070	0.76 ± 0.0090	0.73 ± 0.020	ns	ns	0.02
R. Kidney + R. Adrenal	0.73 ± 0.0180	0.79 ± 0.0070	0.77 ± 0.0150	0.75 ± 0.0160	ns	ns	0.02
Liver	8.51 ± 0.2010	8.84 ± 0.0770	8.86 ± 0.1900	8.73 ± 0.2510	ns	ns	ns
Heart	0.82 ± 0.0140	0.92 ± 0.0530	0.86 ± 0.0120	0.84 ± 0.0180	ns	ns	0.04
Brain	1.95 ± 0.0290	1.98 ± 0.0280	2.02 ± 0.0090	2.01 ± 0.0140	ns	ns	ns
GAS	1.40 ± 0.0670	1.40 ± 0.0270	1.43 ± 0.0310	1.37 ± 0.0210	ns	ns	ns
SOL	0.10 ± 0.0040	0.10 ± 0.0030	0.10 ± 0.0030	0.10 ± 0.0020	ns	ns	ns
TA	0.40 ± 0.0100	0.41 ± 0.0060	0.40 ± 0.0060	0.39 ± 0.0130	ns	ns	ns
EDL	0.12 ± 0.0030	0.12 ± 0.0020	0.13 ± 0.0060	0.13 ± 0.0050	0.02	ns	ns
Left ovary	0.04 ± 0.0020	0.04 ± 0.0030	0.04 ± 0.0020	0.04 ± 0.0030	ns	ns	ns
Right ovary	0.04 ± 0.0020	0.04 ± 0.0020	0.04 ± 0.0020	0.04 ± 0.0020	ns	ns	ns
